# The Book World of Medicine and Science

**Published:** 1899-08-12

**Authors:** 


### The Book World of Medicine and Science.

Chats about the Microscope. By Henry C. Shelley. (London: Scientific Press. 1899. Price 2s. Pp. 101, and numerous illustrations.)

On taking up this little book we were surprised to find no introduction or preface of any kind. The omission, we venture to think, is a pity, for two reasons—first, because no idea is conveyed to the casual reader, one who might very readily pick up such a book at a bookstall—as to the object for which it was written; and secondly, because the individuality of an author is often shown as much by the aptness with which he regulates his subject in accordance with the prescribed lines upon which the book was intended to be written, as by the actual way in which he handles the true subject matter itself. We gather, however, from the first chapter, and curiously, too, from the last, that the idea of the author is to supply a much-felt want, viz., to furnish a little work which shall mainly act as an incentive to the casual reader to take up in a proper manner one of the most fascinating of all pursuits—the use of the microscope. These instruments in the present day are made so cheaply by most of the leading opticians—and not only by the one quoted in the book—that for a few guineas one can be obtained of so useful a form as to afford the owner no end of amusement and instruction provided he only knows how to use it profitably and how to begin. It is just this very point that it appears to us the author has hit off with remarkable skill and judgment. He divides his subject into eight short chapters, which include a somewhat too brief account of the microscope itself, how mounting a specimen is performed, and descriptions of pond-life and of objects frequently met with on the seashore. Botanical subjects, too, do not escape attention, whilst perhaps the most interesting portions of this attractive little book are the remarks, short and concise as they are, on Desmids, Diatonis, Foraminifera, and Rotifers. Readers of this book must not expect to find learned treatises under each of these heads, but merely enough, as the author points out at the end of the last chapter, to induce them, if the spirit is willing, to look the different objects up in works of a more solid nature. It must have been a difficult matter to know what to give and what to omit in compiling a little work of this description, but we must confess surprise at not finding any mention whatever of amœbæ, whilst discussing common objects in pond or ditch-life. The curious movement of their so-called pseudo-podia is a fertile source of amusement even to the skilled microscopist, but it engages and attracts the deepest admiration and attention of almost any novice or beginner in a most pronounced fashion. The book is like all of those issued by the Scientific Press—well printed and neatly bound, and it contains numerous illustrations, mostly of a diagrammatic nature, but fulfilling the object for which they were inserted. We congratulate both the author and the publishers on supplying a want we have often heard expressed: How can we begin to use our microscope? what are we to look for, and where to find it? most books are too deep for us to commence with. This, then, is just what they want, for it is clearly written, well expressed, and contains no severe terminology of any kind.

Golden Rules of Medical Practice. By Arthur Henry Evans, M.D., B.S., F.R.C.S. (Bristol: John Wright and Co. Price 1s.)

This tiny book is No. 4 of the Golden Rules series, and is a very good little collection of aphorisms which one may perfectly fairly call "golden rules" if it pleases one so to do. Most of them are good, some of them touch upon debatable topics, and some are perhaps a little too obvious; still they are nearly all worth remembering and may often prove suggestive.

A Short Practice of Midwifery, Embodying the Treatment Adopted in the Rotunda Hospital, Dublin. By Henry Jellett, B.A., M.D., B.Ch., B.A.O. (Dublin University), F.R.C.P.I. With a preface by W. J. Smily, M.D., F.R.C.P.I. Second Edition. (London: J. and A. Churchill. 1899. Price 6s.)

This is a book which must be considered from two very distinct and separate standpoints; first, how does it teach? and, second, what does it teach? In regard to the first of these we have nothing but praise to give it. The descriptions of the various conditions which may be met with are clear and to the point, the directions given as to their treatment are definite and almost dogmatic. This is as it should be in a book which is largely intended for the use of students, and especially in one which is intended also to be read by midwives going in for their examinations. While plenty of reasons are given to explain and to justify the proceedings recommended, one is never left in doubt as to what to do, nor is one given any excuse for considering the line of treatment an open question. As a teaching book its strong points are the clearness of the descriptions, the appositeness of the illustrations, and the definiteness of the directions; and when we add to this a word in recognition of the excellence of the printing and of the general "get up," we have said all that need be said on this side of the subject.

In considering the adaptability of the book to the wants of students it has, however, to be remembered that it is before all things a handbook of Rotunda methods of treatment, and on the acceptability or otherwise of this treatment must hinge the acceptability of the book itself. We are not going to set ourselves up as judges in regard to matters on which leaders differ, but we must point to one or two of the points in this treatment, the advocacy of which would seem indeed to be one at least of the causes for the production of the work. Two points are especially insisted on. One of these is the use of plugging in cases of external accidental hæmorrhage, the other is the abandonment of the use of chloroform and the substitution of the use of morphine in the treatment of puerperal eclampsia. The case is very ably put; nevertheless, in regard to the first one cannot but see that after all it is the contractility of the uterine muscle, rather than the plug itself, which is the cause of the cessation of the hæmorrhage, in other words that the plugging is but one among many means of setting up uterine contraction. Whether it is the best means will perhaps be questioned. And so in regard to other points. Still the Rotunda methods, taken as a whole, claim to be based upon what must always remain the true test of practice, namely, experience and success. Tables are given showing the nature of all the cases treated in the Rotunda Hospital during the past ten years or so, and also giving details as to the cause of death in every case that ended fatally during that period. It is interesting to note the great differences in both morbidity and fatality in different years, the maternal fatality having in one year mounted up to one in 63·1, and in another sunk to the very low rate of one in 1,524.

### NOTES ON BOOKS.

Aunt Kate's Mothers' Guide.—This is a penny handbook which seems to be intended to teach the modern mother all about babies. We always were sorry for babes, and our pity is in no wise diminished by reading this little book. It may be asked, What can you expect for a penny? But we think that even for a penny something better than this could be produced.

Mr. Frederick Page has put in pamphlet form the results of major amputations treated antiseptically in the Royal Infirmary, Newcastle-upon-Tyne, during the year 1898. For injury there were forty-seven, of which nine died; while for disease there were forty, of which four died. The precise cause of death is given in each case.

